# Sarcomas of fat and bone: a case report

**DOI:** 10.1007/s12672-022-00484-3

**Published:** 2022-04-06

**Authors:** Miriam Beate Honnicke, Lars Tharun, Malte Maria Sieren, Jörg Barkhausen

**Affiliations:** 1 Department of Radiology and Nuclear Medicine, University Hospital Schleswig-Holstein, Campus Lübeck, Ratzeburger Allee 160, 23562 Lübeck, Germany; 2 Institute of Pathology, University Hospital Schleswig-Holstein, Campus Lübeck, Ratzeburger Allee 160, 23562 Lübeck, Germany

**Keywords:** Parosteal osteosarcoma, Osteoliposarcoma, Liposarcoma with osteosarcomatous component, MDM 2, CDK 4

## Abstract

Osteosarcomas are the most common primary malignant bone tumors and are classified by the WHO into several intramedullary and surface subtypes. One of these is the rare parosteal osteosarcoma. Liposarcomas are the second most common soft tissue sarcoma and are classified into several types ranging from intermediate to high grade tumors. In one of our recent patients we found an unusual combination of a parosteal osteosarcoma and a large fatty component, which fluorescence-in-situ-hybridization revealed as liposarcoma. Radiologists, pathologists, and surgeons should consider the possibility of bone and soft tissue malignancies consisting of different components, as this may be of paramount importance for oncologically complete resection.

## Introduction

Osteosarcoma is a common primary malignant bone tumor and is classified into multiple subtypes: conventional (osteoblastic, chondroblastic, fibroblastic), low-grade central, teleangiectatic, small cell, surface (parosteal, periosteal, high-grade surface) and secondary types [1]. Parosteal osteosarcoma (pOS) is a rare low-grade osteosarcoma and accounts for only up to 5% of osteosarcomas, typically manifesting in the 2nd to 4th decade of life. Diagnosis is made and treatment planned through the combination of imaging and histopathologic findings [2].

Liposarcoma is a primary malignant mesenchymal tumor and is also classified by the WHO into subtypes: atypical lipomatous tumor (ALT) / well-differentiated liposarcoma (WDLPS), dedifferentiated liposarcoma (DDLPS), myxoid liposarcoma, pleomorphic liposarcoma and myxoid pleomorphic liposarcoma [1].

In parosteal osteosarcoma as well as ALT/WDLPS and DDLPS two cell cycle oncogenes localized on the chromosomes 12q14-15, murine double minute type 2 (MDM2) and cyclin-dependent kinase 4 (CDK4), are amplified and overexpressed [3, 4], whereas this is not the case in high grade osteosarcoma and pleomorphic liposarcoma [1]. MDM2 and CDK4 can be analyzed immunohistochemically, the detection of an amplification can be further improved by fluorescence in situ hybridization (FISH) analysis which is more sensitive than immunohistochemistry [5].

Our patient showed the typical imaging signs of parosteal osteosarcoma with central to peripheral dense calcification and a cleavage plane, but additionally large radiolucent areas corresponding to fatty tissue in MRI. Fluorescence-in-situ-hybridization revealed MDM2-amplification in the osteosarcomatous as well as the lipomatous component.

The neoplastic nature of a peritumoral fat accumulation may escape detection. Since the rate of recurrence in incomplete or marginal excision is high [6] our aim is to raise awareness to this possibility in order to plan complete oncologic resection.

## Case report

We present the case of a Caucasian woman in her 20 s referred to our Department of Orthopedic surgery. At the time of presentation her BMI was 28.7 with a weight of 88 kg and a height of 175 cm. She had been experiencing pain in her left upper arm and shoulder for 5 months. Physical examination showed a tender, mostly firm mass of the left upper arm, a slightly impaired range of motion and was otherwise unremarkable. Laboratory tests did not show any pathological results. Because of growing discomfort and a slightly impaired range of motion her primary care physician initiated an X-ray and a subsequent MRI.

**Fig. 1:**
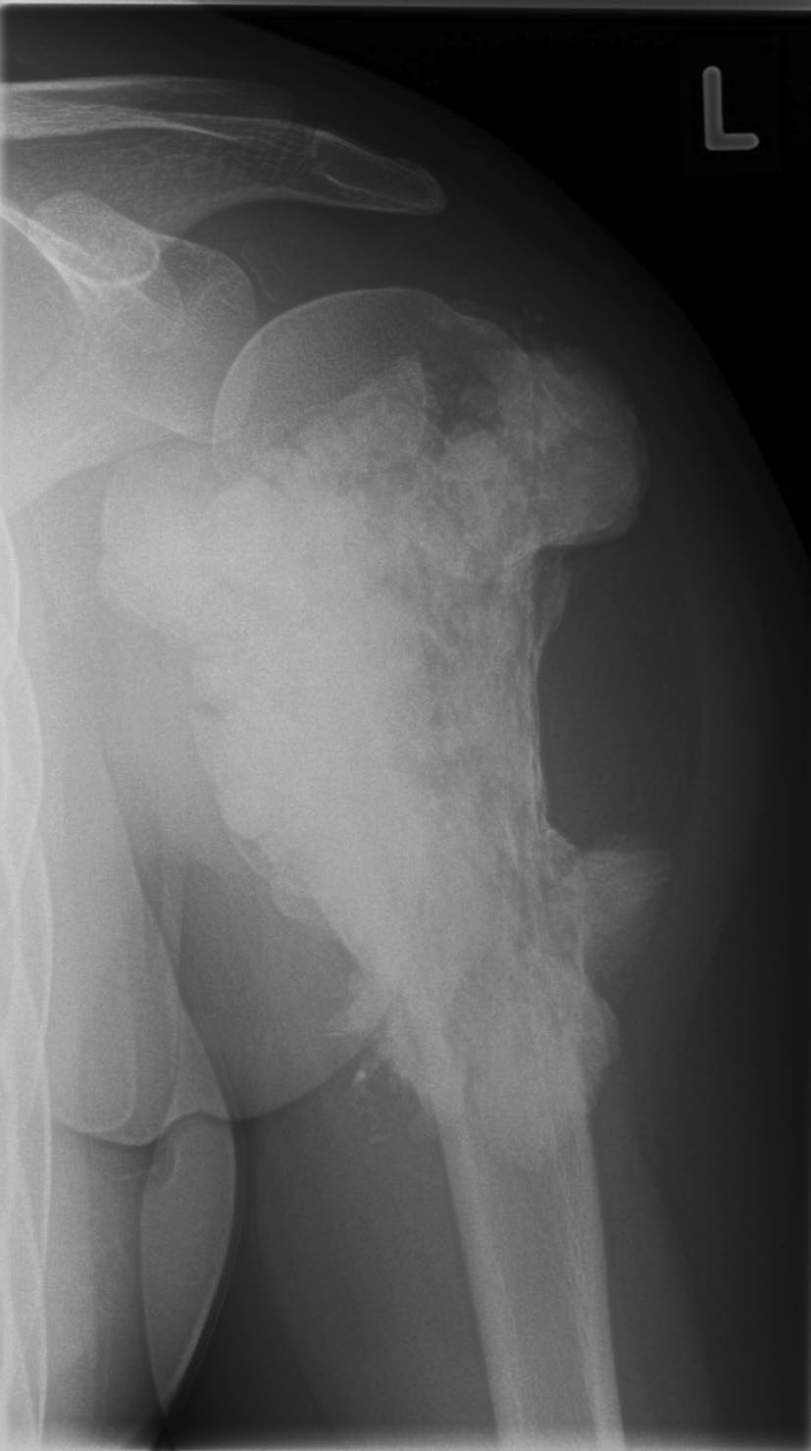
Anterior–posterior radiograph of the left shoulder showing a densely osteoblastic mass encasing the humersu, a cleavage plane and a focal lucency lateral

**Fig. 2:**
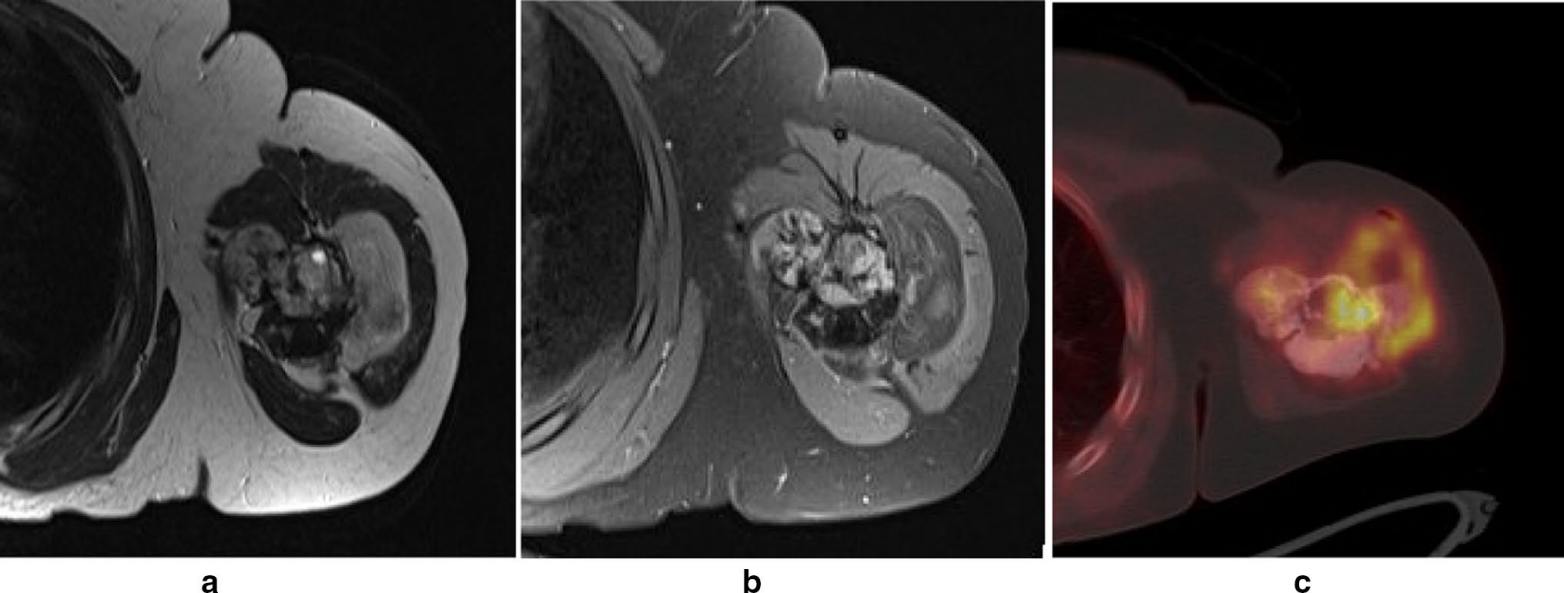
MRI and FDG-PET of the left humerus. **a** T2-weighted image showing the differing signal intensities of the tumor components with low T2 signal in the densely sclerotic part and signal equivalent to fat corresponding to the radiographic lucency. **b** T1-weighted image post contrast with fat suppression confirming the fatty nature of the lateral part of the tumor and showing moderate uptake of gadolinium. **c** FDG-PET-CT image of the tumor depicting a moderate uptake (SUV 4–8)

**Fig. 3:**
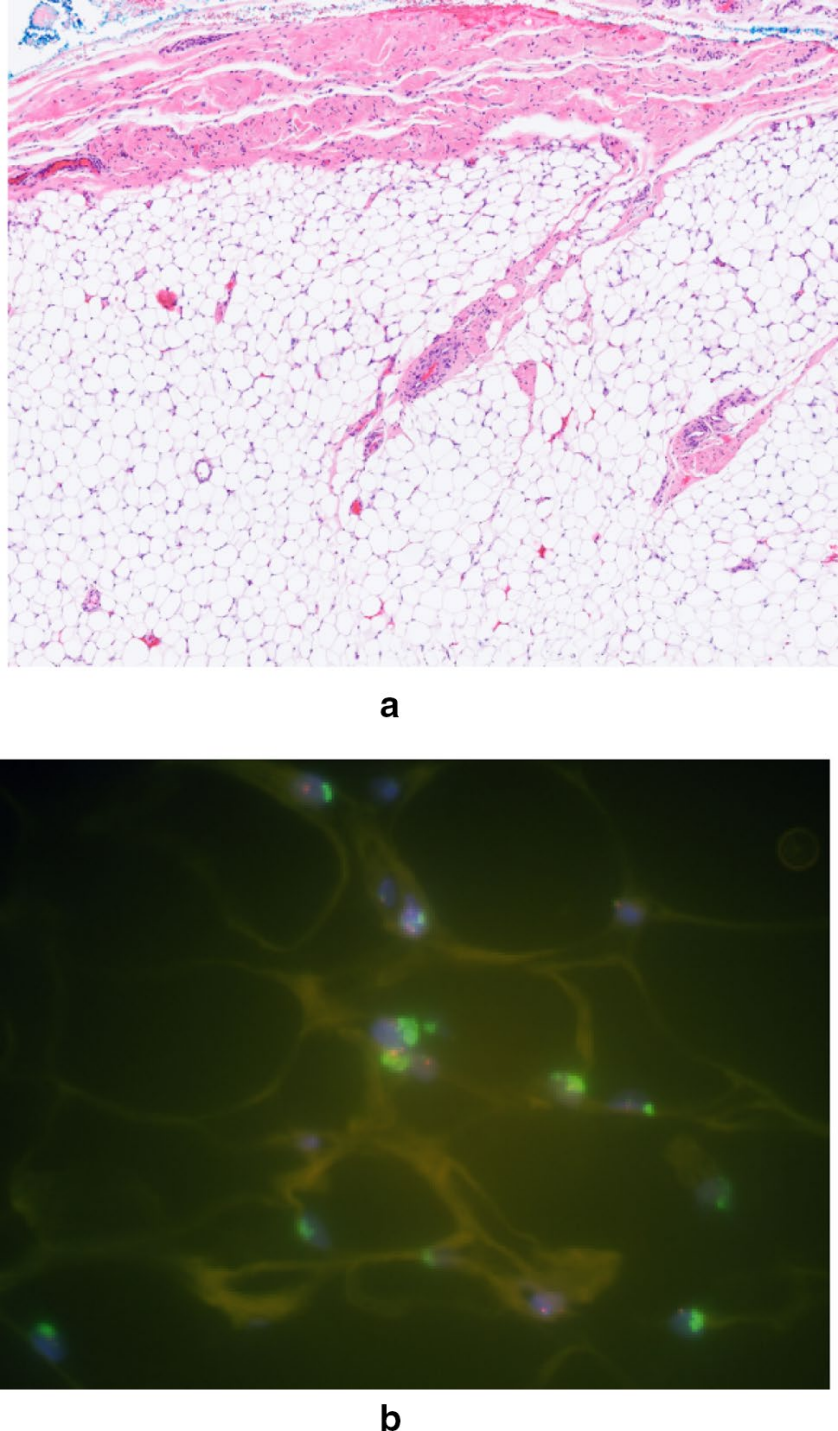
**a** HE stains of the peritumoral fat showing inconspicious lipocytes without atypic vascularization oder atypias. **b** Fluorescence in situ hybridization analysis of the liposarcomatous component with clusters of MDM2 amplification depicted by the brighter signals

**Fig. 4:**
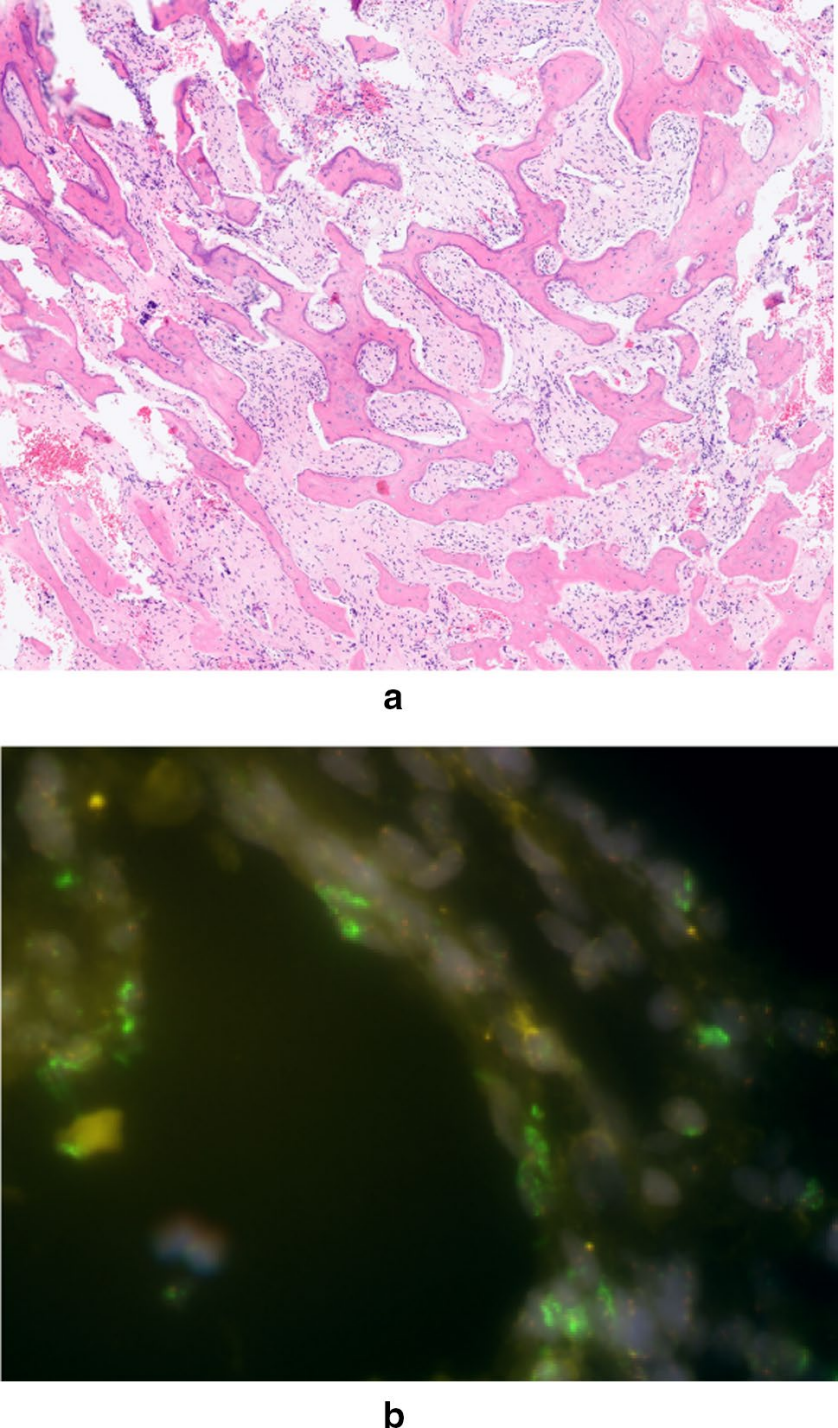
**a** HE stains showing a slightly pleomorphic matrix-forming tumor with prominent capillarization. **b** FISH analysis of the osteosarcomatous component with clusters of MDM2 amplification

The radiograph (Fig. 1) depicted a large mass encasing the proximal left humerus. A thin lucent line next to the greater trochanter indicated a parosteal process. An infiltration into the humerus seemed possible. The matrix was mainly densely osteoblastic, but there was a large focal lucency suggestive of fatty tissue. The suggested diagnosis was parosteal osteosarcoma. The consecutive MRI confirmed considerable amounts of fat within or directly adjacent to the tumor. However, a substantial marrow reaction/infiltration raised doubts about the initial diagnosis and a high-grade osteosarcoma was suspected. Due to the significantly increased risk of metastasis an additional FDG-PET-CT was performed. The examination revealed a moderate focal FDG-uptake (standardized uptake value 4–8) especially at the proximal margins of the lesion. No other foci of pathological uptake were detected, making metastases unlikely (Fig. 2). Based on the imaging findings an open biopsy was carried out. The seven biopsied areas included the extraosseous tumor lateral, proximal intramedullary tissue ventral and dorsal, intramedullary tissue distal, intramedullary fluid and peritumoral fat. The lipomatous tumor component appeared as regular locoregional fatty tissue on conventional microscopy (Fig. 3a). The fluorescence-in-situ-hybridization revealed MDM2-amplification in the intra- and extramedullary as well as the lipomatous component, though, thus confirming a liposarcomatous transdifferentiation of the parosteal osteosarcoma (Fig. 3b). The diagnosis of a low grade parosteal osteosarcoma was confirmed from the extraosseous and the intramedullary matrix-forming component (Fig. 4a and b).

Basal margins of the lesion depicting the low signal of the sclerotic mass adjacent to a large fatty component in the MRI and the moderate FDG-uptake. Biopsies were performed in different areas of the tumor including regions with the highest gadolinium as well as FDG uptake.

## Discussion

A liposarcomatous component in a parosteal osteosarcoma has only been described in two other patients.

Larousserie et al. [7] present a parosteal osteosarcoma in the humerus with peripheral fatty tissue, bone marrow infiltration could not be confirmed or ruled out. MDM2 analysis could not be performed but CDK4 was overexpressed in both tumor components, confirming the sarcomatous nature of each and ruling out metaplasia of the fatty component.

Sohier et al. [8] report a patient with pOS in the tibia first operated on in 1999 and with recurrence in 2011. During the second operation an adipose mass deemed as an incidental lipoma next to the pOS was removed. Since F. Larousserie as one of the authors had encountered a similar entity before, an extensive analysis was performed proving the sarcomatous nature of both components. They discuss three hypotheses about the pathogenesis of this mixed-component tumor, two considering a phenotype switch and a third proposing a common precursor.

Li et al. [9] report one patient with parosteal osteosarcoma of the tibia with two areas of fatty tissue in the tumor in CT imaging. Bone marrow infiltration is not described, an MRI was not performed. The histologic diagnosis was pOS with focal fatty metaplasia, MDM2 and CDK4 were not analyzed. It seems probable that the liposarcomatous component has been missed in this case.

In our own patient the fatty tissue was quite prominent, which initially even raised doubts to the imaging diagnosis of parosteal osteosarcoma. The final diagnosis of two sarcomatous entities or liposarcomatous transdifferentiation of the parosteal osteosarcoma was only possible based on the detection of the MDM2-amplification in fluorescence-in-situ-hybridization.

We summarized all four cases in Table 1. They show that a fatty component in or next to a parosteal osteosarcoma is something radiologists, pathologists and surgeons should look out for. It might well be underdiagnosed because fatty tissue is often unsuspicious on macroscopic observation, imaging and even conventional microcopy. Therefore, histologic analysis should always include FISH-analysis for MDM2 and CDK4.

**Table 1:** Summary of the cases

Larousserie	2011	34	f	Prox humerus parosteal	Peripheral, monofocal	CDK4 positiv, MDM2 not possible	Parosteal osteosarcoma with large fatty component
Li	2018	34	f	Prox tibia parosteal	Peripher, bifocal	“Focal fatty metaplasia”, MDM2 and CDK 4 not done	Parosteal osteosarcoma with fatty component
Sohier	2020	23	f	Prox tibia parosteal	Peripheral, bifocal	MDM2, CDK4 postiv fat, bone not possible	Parosteal osteosarcoma with fatty component
Own case	2021	23	f	Proximal humerus, parosteal	Peripheral, bifocal	MDM2, CDK4 positiv fat and bone	Parosteal osteosarcoma with large fatty component

The coexistence of lipomatous and osseous tissue in sarcomas has often been described and has so far been classified in liposarcomas. Liposarcomas of bone (also titled osteoliposarcomas or malignant mesenchymomas) were initially reported in 1934 [10]. They are quite rare, most are reported to be pleomorphic and as far as they have been analyzed they were reported MDM2 and CDK4 negative [11, 12].

Liposarcomas with osteogenic differentiation are well known and fall in the category of dedifferentiated liposarcoma, the two most common forms of heterologous differentiation in dedifferentiated liposarcoma are myogenic and osteosarcomatous/chondrosarcomatous [13], they show MDM2 amplification.

Whether parosteal osteosarcoma with a liposarcomatous component can be classified into one of the existing subtypes or is a new type of bone tumor as proposed by Larousserie [7] remains open to discussion.

## Summary

Parosteal osteosarcoma combined with perifocal fatty tissue has now been described in four patients. In one case it was judged to be metaplastic fatty tissue adjacent to pOS, but liposarcoma might have been missed due to lack of testing. In our own patient the liposarcoma was only diagnosed by fluorescence-in-situ-hybridization. In three patients the existence of two sarcomatous entities or liposarcomatous transdifferentiation of the parosteal osteosarcoma was proven.

The incidence is probably higher, as peritumoral fatty tissue may not arouse suspicion.

Since the rate of recurrence in incomplete or marginal excision is high, it seems essential to look for a fatty component as part of parosteal osteosarcomas to achieve complete oncologic resection. Even inconspicuous fatty tissue should be biopsied and analyzed for MDM2-amplification, preferably by fluorescence-in-situ-hybridization.

## Abbreviations

LPS: Liposarcoma
ALT/WDLPS: Atypical lipomatous tumor/well differentiated liposarcoma
DDLPS: Dedifferentiated liposarcoma
OS: Osteosarcoma
POS: Parosteal osteosarcoma
c-POS: Conventional parosteal osteosarcoma
DPOS: Dedifferentiated parosteal osteosarcoma
MDM 2, CDK 4: Cell cycle oncogenes: murine double minute type 2, cyclin- dependent kinase 4
FISH: Fluorescence in situ hybridization
